# Exploring the malnutrition status and impact of total parenteral nutrition on the outcome of patients with advanced stage ovarian cancer

**DOI:** 10.1186/s12885-021-08537-6

**Published:** 2021-07-10

**Authors:** Xin Yan, Sanyuan Zhang, Junmei Jia, Jiaolin Yang, Yilai Song, Haoran Duan

**Affiliations:** 1 Department of Drug Clinical Trial, First Hospital of Shanxi Medical University, 85 Jiefangnan Road, Taiyuan, 030001 Shanxi China; 2 Department of Gynecology, First Hospital of Shanxi Medical University, Taiyuan, China; 3 Department of Oncology, First Hospital of Shanxi Medical University, Taiyuan, China; 4 School of Nursing, Shanxi Medical University, Taiyuan, China

**Keywords:** Malnutrition, Total parenteral nutrition, Ovarian cancer, Nutritional risk index, Survival

## Abstract

**Background:**

Ovarian cancer is a common cancer type in women and is often associated with onset of malnutrition. Total parenteral nutrition (TPN) is a nutritional intervention method that has been reported to have controversial effect on cancer patients. In the present retrospective study, we sought to explore the prevalence of malnutrition assessed by the Nutritional Risk Index (NRI) and its association with survival in advanced stage ovarian cancer patients. We also compared the post-operative outcome of the malnourished patients treated with either TPN or conservative management.

**Results:**

A total of 415 patients with advanced stage ovarian cancer were separated into 4 nutrition groups based on the NRI scores. We found that a number of factors were significantly different among the 4 nutrition groups, including age, serum albumin level, BMI and NRI; among which serum albumin level and NRI were identified to be independent predictors of progression-free and overall survival. In the moderately and severely malnourished patients, those who were treated with TPN had significantly shorter hospitalization period, lower serum albumin level and lower BMI after surgery. In addition, serum albumin level, use of TPN and number of patients with complications were closely related to the hospital stay duration.

**Conclusion:**

Malnutrition status is closely associated with survival of advanced stage ovarian cancer patients. These patients may benefit from TPN treatment for reduced hospitalization, especially with the onset of hypoalbuminemia.

## Introduction

Ovarian cancer is a common cancer type among all cancers in women, with over 200 thousand new cases diagnosed worldwide [1]. Due to late presentation and lack of obvious symptoms, ovarian cancer often leads to higher mortality rate compared to other gynecological cancers [2]. Such late diagnosis results in reduced calorie intake and subsequent malnutrition status, where around 50% of patients with ovarian cancer would suffer from malnutrition [3]. Malnutrition can increase the frequency of postoperative complications in cancer patients at advanced stages, resulting in reduced life quality and decreased survival rate [4].

A number of biochemical factors, including serum albumin, transferrin and hemoglobin levels and anthropometric parameters, such as body weight change and body mass index (BMI) have been employed to assess the nutrition status in cancer patients [5–7]. Prealbumin is nowadays often preferred over albumin because of its shorter half-life that detects more rapid changes of the nutritional state [8]. In addition, transferrin has been suggested to be a poor measurement for nutritional status [9] and has been found to be unreliable in the assessment of mild malnutrition in elderly patients [10]. The Nutritional Risk Index (NRI) was developed to assess the nutrition status in malnourished surgical patients [11]. It combines serum albumin level with body weight changes and is a relatively simple and accurate tool to objectively assess nutrition status without the need for any special training [12, 13].

Total parenteral nutrition (TPN) is an intravenous method of feeding nutrients that bypass the gastrointestinal tract. It has been shown to have the potential of improving the malnutrition status in oncology patients [3, 14, 15], whereas no improvement in survival, mitigate toxicity or tumor response rate was observed when TPN [16] or early oral postoperative feeding [17] was added on top of adjuvant therapy. In addition, TPN can be life sustaining for terminally ill ovarian cancer patients [18], despite of its controversial role in other advanced-stage cancers. Current guidelines indicate that when malnourished cancer patients fail to take in nutrients via the gastro-intestinal route for a certain period of time, the TPN should be initiated.

The present retrospective study comprises two main purposes. First, we aim to identify the risk factors that correlate the malnutrition status with survival in patients with advanced ovarian cancer. Second, we aim to compare the outcomes of these patients treated with TPN or conservative management after debulking surgery and bowel resection.

## Methods

### Patients

Data of ovarian cancer patients from 2013 to 2019 were collected from the registered electronic medical records with the institutional approval. The study was approved by the ethical committee of First Hospital of Shanxi Medical University. Included patients had advanced stage (stage 3–4) epithelial ovarian cancer and underwent cytoreductive surgery incorporating bowel resection according to previously published standard (optimal ≤1 cm, suboptimal > 1 cm) [19]. Exclusion criteria include patients who were at stage 1–2 ovarian cancer; patients who did not undergo primary debulking surgery; patients who did not incorporate bowel resection; patients with incomplete treatment and surveillance records. Progression-free survival (PFS) is defined as the time from treatment initiation to the onset of disease progression or patient death. Overall survival (OS) is defined as the time from treatment initiation to patient death.

### Nutrition status assessment

Body weight, BMI and NRI were measured and calculated upon institutional admission. NRI was calculated as [15.19 × serum albumin (g/L)] + [41.7 × current / usual body weight (kg)], where “usual body weight” refers to the normal body weight the patients had prior to illness [11, 20]. Nutrition status was categorized into nourished (NRI > 100), mildly malnourished (NRI 97.5–100), moderately malnourished (83.5–97.4) and severely malnourished (NRI < 83.5).

Subjective global assessment (SGA) was also used to assess patients’ nutritional status upon institutional admission by professional dietitians. During the evaluation, a dietitian reviewed the patients’ medical record, went through all the SGA features and performed a physical body check focusing on loss of subcutaneous fat, muscle wasting, presence of ankle and sacral edema, and ascites. Finally, the patients were ranked into SGA A (nourished), SGA B (moderately malnourished) or SGA C (severely malnourished) as previously described [21].

### Total parenteral nutrition

Initiation of TPN was based on the following criteria: inability of nutrient intake via the gastro-intestinal route due to the surgery and anticipated postoperative oral intake of nutrient exceeding 7 days [22, 23]. To avoid further complications, TPN was terminated when patients resumed their oral intake to obtain the calories needed for their basic body activity. Alternatively, conservative nutritional management (watchful waiting or no TPN) was applied in accordance to physicians’ discretion that precluded TPN.

### Bowel resection type

The ovarian cancer patients were classified into the following clinically defined categories as previously described [24]: 1. Small bowel resection (SBR); 2. Proximal colectomy (PC) alone; 3. Rectosigmoid resection (RSR); 4. Rectosigmoid resection with proximal colectomy (RSR + PC).

### Clinical outcomes

Following factors were documented for patients’ clinical outcomes: pre-operative serum albumin levels, time between surgery and passage of flatus or restoration of bowel function, time between surgery and initiation of TPN, duration of TPN, postoperative complications, length of hospitalization and cases of readmission.

### Statistics

Statistical analysis was performed with SPSS (version 19 for Windows, SPSS Inc., Chicago, IL, USA). Student t test and Mann-Whitney U test were used to compare parametric and nonparametric variables, respectively. Differences between proportions were compared using Fisher’s exact test or χ2 test. Multivariate analysis using Cox regression model adjusted for known prognostic covariates, including surgical FIGO stage, histology and cytoreduction was conducted to analyze factors associated with progression-free and overall survival. *P* < 0.05 was considered as statistically significant.

## Results

### Patient characteristics

A total of 415 patients who had undergone cytoreductive surgery incorporating bowel resection for advanced epithelial ovarian cancer were included in the present study. Patients were separated into nourished, mildly malnourished, moderately malnourished and severely malnourished groups based on the NRI score. The medians of PFS and OS were 89 days (range 10–200) and 227 days (range 83–396), respectively. Significant difference was observed for both PFS and OS among the 4 patient groups (Table 1). Patients’ age was found to be significantly different among the 4 nutrition groups (Table 1). Majority of the patients had stage 3 cancer mainly with serous histology and demonstrated optimal cytoreduction (Table 1). Time of flatus passage or restoration of bowel function and time of hospitalization were found to be similar among the 4 groups (Table 1). Serum albumin level and BMI at admission and after surgery were all significantly lower as the malnutrition status getting worse (Table 1). We also grouped the patients according to SGA and observed similar outcome (Table 2).

**Table 1:** Characteristics of patients of the 4 nutrition groups categorized based on NRI

	Total patients	Nourished (*n* = 96)	Mildly malnourished (*n* = 122)	Moderately malnourished (*n* = 101)	Severely Malnourished (*n* = 96)	*P* value
Age (years, median, range)	50 (25–75)	54.5 (25–75)	46 (25–75)	54 (25–74)	45 (26–75)	0.002
Surgical FIGO stage						0.08
Stage 3 (n, %)	348 (83.9%)	81 (84.4%)	102 (83.6%)	84 (83.2%)	81 (84.4%)	
Stage 4 (n, %)	67 (16.1%)	15 (15.6)	20 (16.4%)	17 (16.8%)	15 (15.6%)	
Histology						0.998
Serous (n, %)	330 (79.5%)	77 (80.2%)	97 (79.5%)	81 (80.2%)	75 (78.1%)	
Endometrioid (n, %)	48 (11.6%)	11 (11.5%)	16 (13.1%)	9 (8.9%)	12 (12.5%)	
Mixed (n, %)	15 (3.6%)	3 (3.1%)	4 (3.3%)	5 (5.0%)	3 (3.1%)	
Clear cell (n, %)	12 (2.9%)	3 (3.1%)	3 (2.5%)	3 (3.0%)	3 (3.1%)	
Other (n, %)	10 (2.4%)	2 (2.1%)	2 (1.6%)	3 (3.0%)	3 (3.1%)	
Cytoreduction						0.51
Optimal (n, %)	350 (84.3%)	83 (86.5%)	105 (86.1%)	80 (79.2%)	82 (85.4%)	
Sub-optimal (n, %)	65 (15.7%)	13 (13.5%)	17 (13.9%)	21 (20.8%)	14 (14.6%)	
Days to flatus (median, range)	7 (4–10)	7.5 (4–10)	7 (4–10)	7 (4–10)	6.5 (4–10)	0.472
Days of hospitalization (median, range)	12 (5–20)	12 (5–20)	12 (5–20)	13 (5–20)	13 (5–20)	0.52
Serum albumin (g/L) (median, range)	4 (1–6)	5.1 (4–6)	4.6 (3–6)	3 (1–5)	2.45 (1–4)	< 0.001
BMI at admission (median, range)	23.9 (16–40)	27.05 (16.3–39.8)	27.8 (16.1–40)	22.9 (16.1–29.8)	20 (16–25)	< 0.001
BMI after surgery (median, range)	23.6 (15.2–35)	25.05 (15.2–35)	26.4 (16.1–33)	23.7 (15.2–30)	21.05 (16–26)	< 0.001
NRI at admission (median, range)	97.7 (75.1–108)	104.75 (100–108)	98.8 (97.5–100)	91 (83.5–97.4)	80.1 (75.1–83.3)	< 0.001
PFS (days, median, range)	89 (10–200)	108 (23–200)	108.5 (20–179)	86 (10–150)	67.5 (10–120)	< 0.001
OS (days, median, range)	227 (83–396)	247.5 (102–396)	237 (104–380)	224 (100–328)	204.5 (83–298)	< 0.001

**Table 2:** Characteristics of patients of the 4 nutrition groups categorized based on SGA

	Nourished (*n* = 202)	Moderately malnourished (*n* = 115)	Severely malnourished (*n* = 98)	*P* value
Age (years, median, range)	50 (25–75)	54 (25–74)	45 (26–75)	0.2268
Surgical FIGO stage				0.0745
Stage 3 (n, %)	175 (86.6%)	98 (85.2%)	75 (76.5%)	
Stage 4 (n, %)	27 (13.4%)	17 (14.8%)	23 (23.5%)	
Histology				0.7059
Serous (n, %)	166 (82.2%)	89 (77.4%)	75 (76.5%)	
Endometrioid (n, %)	22 (10.9%)	15 (13.0%)	11 (11.2%)	
Mixed (n, %)	6 (3.0%)	5 (4.3%)	4 (4.1%)	
Clear cell (n, %)	5 (2.5%)	4 (3.5%)	2 (2.0%)	
Other (n, %)	3 (1.5%)	2 (1.7%)	5 (5.1%)	
Cytoreduction				0.0793
Optimal (n, %)	177 (87.6%)	97 (84.3%)	76 (77.6%)	
Sub-optimal (n, %)	25 (12.4%)	18 (15.7%)	22 (22.4%)	
Days to flatus (median, range)	7 (4–10)	7 (4–10)	6.5 (4–10)	0.7305
Days of hospitalization (median, range)	12 (5–20)	13 (5–20)	12.5 (5–20)	0.673
Serum albumin (g/L) (median, range)	4.9 (3–6)	3.3 (1–5.9)	2.45 (1–4.8)	< 0.001
BMI at admission (median, range)	27.9 (16.1–40)	22.8 (16.1–39.6)	20.15 (16–29.3)	< 0.001
BMI after surgery (median, range)	25.9 (15.2–35)	23.8 (15.2–30.9)	21.05 (16–26)	< 0.001
NRI at admission (median, range)	100 (97.5–108)	92 (83.5–99.9)	80.15 (75.5–91.4)	< 0.001
PFS (days, median, range)	108 (20–200)	90 (10–179)	67.5 (10–120)	< 0.001
OS (days, median, range)	240.5 (102–396)	227 (100–357)	207.5 (983–298)	< 0.001

### Factors predicting patients’ survival outcome

All patients were followed for at least 6 months, with a median of 24 months ranged from 6 to 48 months. During the follow-up period, 147 patients have passed away as a result of the ovarian cancer. We picked the factors that were significantly different among the 4 nutrition groups and performed univariate and multivariate Cox regression analysis for progression-free and overall survival of the patients. The overall survival does not include perioperative mortality. In the univariate analysis focusing on individual variables, we found that patients’ age, serum albumin level and NRI measured at admission were significantly associated with the progression-free survival of the patients (Table 3). In the multivariate analysis, the 3 factors were found to be independent predictors of poor progression-free survival after adjustment for BMI at admission and after surgery (Table 3). Similar outcome was observed for the overall survival status, except that age was not identified as an independent predictor for poor overall survival (Table 4).

**Table 3:** Univariate and multivariate Cox regression model analysis of factors predicting progression-free survival of the patients with advanced stage ovarian cancer

	Univariate analysis			Multivariate analysis		
	HR	95% CI	*P* value	HR	95% CI	*P* value
Days of hospitalization	Reference			Reference		
Age	3.065	1.948–4.129	0.041	3.415	2.477–4.025	0.037
Serum albumin	7.589	5.287–9.046	< 0.001	8.147	6.245–9.898	< 0.001
BMI at admission	1.258	1.005–1.429	0.225			
BMI after surgery	1.345	1.059–1.593	0.397			
NRI at admission	1.979	1.589–2.343	0.035	2.041	1.426–2.846	0.037

**Table 4:** Univariate and multivariate Cox regression model analysis of factors predicting overall survival of the patients with advanced stage ovarian cancer

	Univariate analysis			Multivariate analysis		
	HR	95% CI	*P* value	HR	95% CI	*P* value
Days of hospitalization	Reference			Reference		
Age	2.957	2.125–3.674	0.042	2.845	2.147–3.876	0.059
Serum albumin	6.248	4.856–8.584	< 0.001	7.134	5.034–9.014	< 0.001
BMI at admission	1.568	1.241–1.958	0.576			
BMI after surgery	1.638	1.148–1.974	0.423			
NRI at admission	2.587	1.845–2.936	0.033	2.668	1.954–3.547	0.036

### TPN impact on malnutrition

Next, we concentrated on the patients in the moderately and severely malnourished groups, and classified them into two new groups based on whether they had the TPN or the conservative nutritional management after surgery. There were 57 patients (28.9%) who received TPN and 140 patients (71.1%) who received the conservative management (Table 5). In general, no significant differences were observed in terms of age, time of flatus passage or restoration of bowel function, BMI and NRI at admission, type of bowel resection and numbers of complications and readmissions (Table 5). Patients treated with TPN had significantly less hospitalization time, lower serum albumin level and smaller BMI values after surgery (Table 5). For the TPN treated patients, mean time to initiate the nutrition intervention was 2.8 ± 1.5 days, with a mean duration of 3.3 ± 1.6 days (Table 5).

**Table 5:** Characteristics of the moderately and severely malnourished patients treated with either TPN or conservative management. TPN, total parenteral nutrition; BMI, body mass index; NRI, nutritional risk index; SBR, small bowel resection; PC, proximal colectomy; RSR, Rectosigmoid resection

	TPN (*n* = 57)	Conservative management (*n* = 140)	*P* value
Age (years, median, range)	47 (25–75)	50 (25–75)	0.6
Days to flatus (median, range)	7 (4–10)	7 (4–10)	0.09
Days of hospitalization (median, range)	11 (5–20)	13 (5–20)	0.02
Serum albumin (g/L) (median, range)	1.4 (1–1.9)	3.2 (2–5)	< 0.001
BMI at admission (median, range)	61.6 (16–29.4)	20.9 (16.1–29.8)	0.9
BMI after surgery (median, range)	20.8 (15.6–29.4)	22.15 (15.2–30)	0.048
NRI at admission (median, range)	81.9 (75.2–97.3)	84.3 (75.1–97.4)	0.13
Days between surgery and TPN initiation (median, range)	3 (1–5)		
Duration of TPN (median, range)	3 (1–6)		
Bowel resection			
SBR (n, %)	13 (22.8%)	32 (22.9%)	0.998
PC (n, %)	14 (24.6%)	34 (24.3%)	
RSR (n, %)	12 (21.1%)	31 (22.1%)	
RSR + PC (n, %)	18 (31.6%)	43 (30.7%)	
Complications (n, %)	4 (7.0%)	11 (7.9%)	0.84
Readmissions (n, %)	3 (5.3%)	8 (5.7%)	0.9

There were 4 patients in the TPN group and 11 patients from the conservative management group experienced post-operative complications (Table 5). In the TPN group, two patients experienced small bowel obstruction and were treated with bowel rest and/or intubation. The other two patients suffered entero-cutaneous fistula and was surgically managed by colon resection and anastomosis. Except for one patient that suffered small bowel obstruction, all the other three patients were readmitted to the hospital. In the conservative management group, 8 patients suffered surgical site infections, all of whom were readmitted for drainage and anti-biotic treatment. The other three patients experienced small bowel obstruction and were all successfully managed by bowel rest.

Finally, we performed univariate and multivariate Cox regression analysis to individually and combinatorically analyze the impact of many factors on hospital stay duration. We found that lower serum albumin, use of TPN and numbers of complications were all closely related to the duration of hospital stay (Table 6).

**Table 6:** Univariate and multivariate Cox regression model analysis of factors predicting the hospital stay duration of the moderately and severely malnourished patients with advanced stage ovarian cancer

	Univariate analysis			Multivariate analysis		
	HR	95% CI	*P* value	HR	95% CI	*P* value
BMI at admission	Reference			Reference		
Age	1.052	0.921–1.232	0.658			
SBR	1.248	0.998–1.367	0.543			
PC	1.154	0.945–1.269	0.387			
RSR	1.335	1.116–1.587	0.378			
RSR + PC	1.268	1.025–1.358	0.584			
Days to flatus	1.898	1.145–2.259	0.112			
Serum albumin (g/L)	5.287	2.458–7.025	< 0.001	6.954	3.876–8.023	< 0.001
Use of TPN	3.848	3.045–4.147	0.025	4.652	3.249–5.248	0.031
Number of complications	2.987	2.154–3.325	0.032	3.256	2.512–4.689	0.035

## Discussion

NRI has been used in several studies to assess the nutritional status in relation to post-operative complications, hospitalization and disease morbidity in patients who underwent surgery [25–27]. For cancer patients, a few prognostic scoring systems have been employed to assess their nutritional status, including Prognostic Nutritional Index (PNI) [28], Patient-generated Subjective Global Assessment (PG-SGA) [15]. However, these tools are limited by their subjective assessment nature that may require experienced assessors to obtain consistent and reliable results [29], despite that there was a study showing their accuracy in predicting malnutrition and survival status in gynecological cancer patients [15]. On the other hand, NRI is based on two objective parameters, body weight and serum albumin to make the assessment. In addition, the “usual body weight” value used in the formula can be related to a history of recent weight loss, a factor that has been previously implicated in increased mortality rate in elderly people [30].

With the aid of the NRI scoring system, among the 415 included patients nearly half of them (197 out of 415) were diagnosed to be moderately or severely malnourished. Consistent with previous studies [31], we found that BMI values measured both at admission and after surgery were not correlated with overall survival of the patients, although they are significantly different among the nutritional groups. One possible cause for the differential BMI after surgery is postoperative fluid gain. In fact, perioperative fluid balance has been suggested to be a significant predictor for postoperative complications in patients with advanced epithelial ovarian cancer [32]. This might explain the reduced level of serum albumin in poorly nourished patients due to the dilutional effect, although further analysis to distinguish between fluid and fat/lean body mass gain is required to validate this reasoning. In addition, serum albumin and NRI score were found to be independent predictors for progression-free and overall survival of ovarian patients, which is also in line with previous findings [15, 31]. Surprisingly, patients’ age was found to be significantly different among the four nutritional groups, where the mildly and severely malnourished patients are younger than the nourished and moderately nourished patients. Moreover, age was also identified as an independent predictor for patients’ progression-free survival. This could be due to the narrower age range of the included patients in the present study as compared to previous studies [15, 31].

The use of TPN to treat post-operative patients remains controversial, where some studies indicate that there is too little clinical benefit to warrant the nutritional intervention [16, 33]. On the other hand, another study has demonstrated significantly improved median survival for terminally ill ovarian cancer patients who received TPN [22]. In the present study, we found that TPN treatment can reduce the time of hospitalization as previously reported [3, 14, 15], but had no impact on time to restoration of bowel function and number of post-operative complications, which was suggested to be otherwise in a previous study [34]. This could be due to the difference in the analyzed population between the two studies. However, in line with this study [34], we also found that serum albumin level was significantly lower in the TPN treated patients. Serum albumin often reflects elevation of systemic immune response and metabolism status as a result of traumatic injury and has been shown to be a significant predictor for operative morbidity and surgical outcome [35, 36]. Therefore, management of severe hypoalbuminemia might be critical for better post-operative outcome.

The present study has several limitations that needs to be noted when interpreting the results. Given the retrospective nature of the study, a potential of selection bias and incomplete data collection may affect the study outcome. Without prospective stratification and planned randomization, the comparison among different nutrition and treatment groups may be impaired by significant biases. Also, the patient population was selected from a single institution, which may also impinge on the data analysis. In addition, we did not include patients who did not have bowel surgery, which is a potential factor that might affect patients’ nutritional status. Lastly, although our selection criteria for TPN treatment is in line with another previous study [34], it is slightly different from other reported guidelines [37]. On the other hand, our study is the first one to investigate the malnutrition status and impact of TPN on the outcome of patients with advanced stage ovarian cancer in a Chinese population. Given that many of the previous studies are also limited by their retrospective nature and small sample size, findings of our study will add confidence on the present view of TPN usage for cancer patients.

In summary, we have shown that almost half of the patients with advanced stage ovarian cancer suffered from moderate or severe malnutrition. Serum albumin level and NRI score at admission were significantly associated with the poor survival outcome. In addition, lower serum albumin, use of TPN and numbers of complications were all closely related to the length of hospital stay, suggesting that TPN should be considered as a positive treatment method for advanced stage ovarian cancer patients.

## Data Availability

Data sharing is not applicable to this article as no datasets were generated or analysed during the current study.

## References

[1] Ferlay J, Shin HR, Bray F, Forman D, Mathers C, Parkin DM (2010). Estimates of worldwide burden of cancer in 2008: GLOBOCAN 2008. Int J Cancer.

[2] Bandera EV, Kushi LH, Rodriguez-Rodriguez L (2009). Nutritional factors in ovarian cancer survival. Nutr Cancer.

[3] Balogun N, Forbes A, Widschwendter M, Lanceley A (2012). Noninvasive nutritional management of ovarian cancer patients: beyond intestinal obstruction. Int J Gynecol Cancer.

[4] Garth AK, Newsome CM, Simmance N, Crowe TC (2010). Nutritional status, nutrition practices and post-operative complications in patients with gastrointestinal cancer. J Hum Nutr Diet.

[5] Campbell PT, Newton CC, Dehal AN, Jacobs EJ, Patel AV, Gapstur SM (2012). Impact of body mass index on survival after colorectal cancer diagnosis: the Cancer prevention study-II nutrition cohort. J Clin Oncol.

[6] Correira Pereira MA, Santos CA, Almeida Brito J, Fonseca J (2014). Scored patient-generated subjective global assessment, albumin and transferrin for nutritional assessment of gastrostomy fed head or neck cancer patients. Nutr Hosp.

[7] Unal D, Orhan O, Eroglu C, Kaplan B (2013). Prealbumin is a more sensitive marker than albumin to assess the nutritional status in patients undergoing radiotherapy for head and neck cancer. Contemp Oncol.

[8] Keller U. Nutritional Laboratory Markers in Malnutrition. J Clin Med. 2019;8(6):775. 10.3390/jcm8060775. 10.3390/jcm8060775 PMC6616535 31159248

[9] Roza AM, Tuitt D, Shizgal HM (1984). Transferrin--a poor measure of nutritional status. JPEN J Parenter Enteral Nutr.

[10] Sergi G, Coin A, Enzi G, Volpato S, Inelmen EM, Buttarello M, Peloso M, Mulone S, Marin S, Bonometto P (2006). Role of visceral proteins in detecting malnutrition in the elderly. Eur J Clin Nutr.

[11] Buzby GP, Knox LS, Crosby LO, Eisenberg JM, Haakenson CM, McNeal GE (1988). Study protocol: a randomized clinical trial of total parenteral nutrition in malnourished surgical patients. Am J Clin Nutr.

[12] Cereda E, Limonta D, Pusani C, Vanotti A (2006). Assessing elderly at risk of malnutrition: the new geriatric nutritional risk index versus nutritional risk index. Nutrition..

[13] Oh CA, Kim DH, Oh SJ, Choi MG, Noh JH, Sohn TS, Bae JM, Kim S (2012). Nutritional risk index as a predictor of postoperative wound complications after gastrectomy. World J Gastroenterol.

[14] Baker J, Janda M, Graves N, Bauer J, Banks M, Garrett A, Chetty N, Crandon AJ, Land R, Nascimento M, Nicklin JL, Perrin LC, Obermair A (2015). Quality of life after early enteral feeding versus standard care for proven or suspected advanced epithelial ovarian cancer: results from a randomised trial. Gynecol Oncol.

[15] Laky B, Janda M, Kondalsamy-Chennakesavan S, Cleghorn G, Obermair A (2010). Pretreatment malnutrition and quality of life - association with prolonged length of hospital stay among patients with gynecological cancer: a cohort study. BMC Cancer.

[16] Barber MD, Fearon KC, Delmore G, Loprinzi CL (1998). Should cancer patients with incurable disease receive parenteral or enteral nutritional support?. Eur J Cancer.

[17] Minig L, Biffi R, Zanagnolo V, Attanasio A, Beltrami C, Bocciolone L, Botteri E, Colombo N, Iodice S, Landoni F, Peiretti M, Roviglione G, Maggioni A (2009). Early oral versus "traditional" postoperative feeding in gynecologic oncology patients undergoing intestinal resection: a randomized controlled trial. Ann Surg Oncol.

[18] Howard L, Heaphey L, Fleming CR, Lininger L, Steiger E (1991). Four years of north American registry home parenteral nutrition outcome data and their implications for patient management. JPEN J Parenter Enteral Nutr.

[19] Chi DS, Eisenhauer EL, Lang J, Huh J, Haddad L, Abu-Rustum NR, Sonoda Y, Levine DA, Hensley M, Barakat RR (2006). What is the optimal goal of primary cytoreductive surgery for bulky stage IIIC epithelial ovarian carcinoma (EOC)?. Gynecol Oncol.

[20] Kuzu MA, Terzioglu H, Genc V, Erkek AB, Ozban M, Sonyurek P (2006). Preoperative nutritional risk assessment in predicting postoperative outcome in patients undergoing major surgery. World J Surg.

[21] Detsky AS, McLaughlin JR, Baker JP, Johnston N, Whittaker S, Mendelson RA (1987). What is subjective global assessment of nutritional status?. J Parenter Enteral Nutr.

[22] Brard L, Weitzen S, Strubel-Lagan SL, Swamy N, Gordinier ME, Moore RG, Granai CO (2006). The effect of total parenteral nutrition on the survival of terminally ill ovarian cancer patients. Gynecol Oncol.

[23] Gerardi MA, Santillan A, Meisner B, Zahurak ML, Montes TPD, Giuntoli RL (2008). A clinical pathway for patients undergoing primary cytoreductive surgery with rectosigmoid colectomy for advanced ovarian and primary peritoneal cancers. Gynecol Oncol.

[24] Rettenmaier MA, Abaid LN, Brown JV, Mendivil AA, Micha JP, Goldstein BH (2014). The incidence of postprandial nausea and nutritional regression in gynecologic cancer patients following intestinal surgery: a retrospective cohort study. Int J Surg.

[25] Abd-El-Gawad WM, Abou-Hashem RM, El Maraghy MO, Amin GE (2014). The validity of geriatric nutrition risk index: simple tool for prediction of nutritional-related complication of hospitalized elderly patients. Comparison with mini nutritional assessment. Clin Nutr.

[26] Aziz EF, Javed F, Pratap B, Musat D, Nader A, Pulimi S, Alivar CL, Herzog E, Kukin ML (2011). Malnutrition as assessed by nutritional risk index is associated with worse outcome in patients admitted with acute decompensated heart failure: an ACAP-HF data analysis. Heart Int.

[27] Kyle UG, Pirlich M, Schuetz T, Lochs H, Pichard C (2004). Is nutritional depletion by nutritional risk index associated with increased length of hospital stay? A population-based study. JPEN J Parenter Enteral Nutr.

[28] Ishizuka M, Oyama Y, Abe A, Tago K, Tanaka G, Kubota K (2014). Prognostic nutritional index is associated with survival after total gastrectomy for patients with gastric cancer. Anticancer Res.

[29] Kobayashi I, Ishimura E, Kato Y, Okuno S, Yamamoto T, Yamakawa T, Mori K, Inaba M, Nishizawa Y (2010). Geriatric nutritional risk index, a simplified nutritional screening index, is a significant predictor of mortality in chronic dialysis patients. Nephrol Dial Transplant.

[30] Omran ML, Morley JE (2000). Assessment of protein energy malnutrition in older persons, part I: history, examination, body composition, and screening tools. Nutrition..

[31] Yim GW, Eoh KJ, Kim SW, Nam EJ, Kim YT (2016). Malnutrition identified by the nutritional risk index and poor prognosis in advanced epithelial ovarian carcinoma. Nutr Cancer.

[32] Hasselgren E, Hertzberg D, Camderman T, Bjorne H, Salehi S (2021). Perioperative fluid balance and major postoperative complications in surgery for advanced epithelial ovarian cancer. Gynecol Oncol.

[33] Parenteral nutrition in patients receiving cancer chemotherapy. American College of Physicians. Ann Intern Med. 1989;110(9):734–6. 10.7326/0003-4819-110-9-734 2494922

[34] Mendivil AA, Rettenmaier MA, Abaid LN, Brown JV, Mori KM, Goldstein BH (2017). The impact of total parenteral nutrition on postoperative recovery in patients treated for advanced stage ovarian cancer. Arch Gynecol Obstet.

[35] Ataseven B, du Bois A, Reinthaller A, Traut A, Heitz F, Aust S, Prader S, Polterauer S, Harter P, Grimm C (2015). Pre-operative serum albumin is associated with post-operative complication rate and overall survival in patients with epithelial ovarian cancer undergoing cytoreductive surgery. Gynecol Oncol.

[36] Ryan AM, Hearty A, Prichard RS, Cunningham A, Rowley SP, Reynolds JV (2007). Association of hypoalbuminemia on the first postoperative day and complications following esophagectomy. J Gastrointest Surg.

[37] Braga M, Ljungqvist O, Soeters P, Fearon K, Weimann A, Bozzetti F, ESPEN (2009). ESPEN guidelines on parenteral nutrition: surgery. Clin Nutr.

